# Cyclophilin A Impairs Efferocytosis and Accelerates Atherosclerosis by Overexpressing CD 47 and Down-Regulating Calreticulin

**DOI:** 10.3390/cells10123598

**Published:** 2021-12-20

**Authors:** Vinitha Anandan, Thushara Thulaseedharan, Aishwarya Suresh Kumar, Karthika Chandran Latha, Amjesh Revikumar, Ajit Mullasari, Chandrasekharan C. Kartha, Abdul Jaleel, Surya Ramachandran

**Affiliations:** 1 Cardiovascular Diseases and Diabetes Biology, Rajiv Gandhi Centre for Biotechnology, Thiruvananthapuram 695014, Kerala, India; vinithaa@rgcb.res.in (V.A.); thushara@rgcb.res.in (T.T.); sureshkumaraishwarya@gmail.com (A.S.K.); karthikac@rgcb.res.in (K.C.L.); amjeshr@rgcb.res.in (A.R.); jaleel@rgcb.res.in (A.J.); 2 Ph.D. Program, Manipal Academy of Higher Education, Udupi 576104, Manipal, India; 3 Madras Medical Mission, Institute of Cardio-Vascular Diseases, Mogappair, Chennai 600037, Tamil Nadu, India; icvddoctors@mmm.org; 4 Department of Neurology, Amrita Institute of Medical Sciences, Amrita Vishwa Vidyapeetham, Kochi 682041, Kerala, India; cckartha@gmail.com

**Keywords:** atherosclerosis, macrophages, efferocytosis, apoptosis, cyclophilin A, CD 47, calreticulin, ATP binding cassette subfamily A, member 1

## Abstract

Impairment of efferocytosis in apoptotic macrophages is a known determinant of the severity of atherosclerosis and the vulnerability of plaques to rupture. The precise mechanisms involved in impaired efferocytosis are unclear. Given the well-recognized role of the inflammatory cytokine cyclophilin A (Cyp A) in modulating several atherogenic mechanisms in high-glucose primed monocytes, we investigated the role of Cyp A in macrophage efferocytosis. The efficiency of efferocytosis in RAW 264.7 macrophages grown in vitro and primed with cyclophilin A was assessed using flow cytometry and confocal assays. Cholesterol content in cells was measured using cell-based cholesterol efflux assay. Proteomic analysis and bioinformatics tools were employed to decipher the link between cyclophilin A and the known ligand receptors involved in efferocytosis. Cyclophilin A was found to impair efferocytosis in apoptotic macrophages by reducing ABCA1-mediated cholesterol efflux in foam cells derived from macrophages. Cyclophilin A-primed macrophages showed an increase in expression of the *don’t-eat-me* signal CD 47 and a decrease in the expression of the *eat-me* signal, calreticulin. Phagocytosis was restored upon silencing of cyclophilin A. New Zealand white rabbits were fed a high-fat diet, and lesions in their aortae were analyzed histologically for evidence of atherosclerosis and the expression of Cyp A, CD 47 and calreticulin, the ligand receptor involved in efferocytosis. Gene and protein expressions in aortae and macrophages were analyzed by real-time PCR and Western blotting. Cyclophilin A, via its effects on the expression of CD 47 and calreticulin, impairs efferocytosis in apoptotic macrophages. Together with its impact on cholesterol efflux from macrophages, these effects can amplify other mechanisms of Cyp A in accelerating the progression of atherosclerosis.

## 1. Introduction

Cyclophilin A (Cyp A) is a universally distributed protein that exists in both intracellular and extracellular forms [1,2]. Several cells, such as endothelial cells, vascular smooth muscle cells and platelets, secrete Cyp A in response to increased oxidative stress [3]. Cyp A is known to mediate the progression of atherosclerosis by increasing adhesion, transmigration and differentiation of monocytes, as well as the formation of foam cells [2]. In high-glucose conditions, Cyp A can induce apoptosis of macrophages [4]. It is possible that the increased incidence of apoptotic cells is due to inadequate clearance of apoptotic cells in the vascular lumen, a process called efferocytosis. Efferocytosis involves several sequential events, including finding of apoptotic cells by phagocytes, recognition of apoptotic cells (ACs) by phagocytic receptors, activation of intracellular signaling cascades with cytoskeletal rearrangements for AC engulfment and post-engulfment processes for phagosome maturation and AC degradation [5]. These mechanisms are regulated by several signal molecules, such as ‘*find-me*’, ‘*eat-me*’ and ‘*don’t-eat-me*’ signals. Cells undergoing apoptosis express molecules such as ‘find-me’ signals to attract phagocytes, whereas, dying cells also express ‘*eat-me*’ signals on the cell surface to indicate they should be engulfed by macrophages. The best-characterized ‘*eat-me*’ signal is phosphatidylserine on the cell surface of ACs. Healthy cells display ‘*don’t-eat-me*’ signals, such as CD 47 and CD 31, on the cell surface to avoid efferocytosis. The prompt and efficient clearance of apoptotic cells by phagocytosis is essential to maintenance of tissue homeostasis and prevention of secondary necrosis [6]. The necrotic core is the hallmark of the vulnerable atherosclerotic plaque. Although apoptotic cells are cleared quickly in almost all other tissue beds, their removal appears to be significantly impaired in diseased blood vessels [7]. Macrophages containing abundant apoptotic material are detected in plaques, supporting that efferocytosis occurs in atherosclerosis [8].

We report here that Cyp A impairs efferocytosis in apoptotic macrophages associated with atherosclerotic lesions. Impaired efferocytosis, together with an increase in macrophage apoptosis, can modulate the severity of atherosclerotic lesion. This mechanism could be important for rapid progression of atherosclerotic lesions in type 2 diabetes mellitus, where circulating levels of Cyp A are elevated [9].

## 2. Materials and Methods

### 2.1. In Vitro Culture Model

RAW 264.7 macrophages, obtained from American Type Culture Collection (ATCC^®^ TIB-71™ USA, were maintained in a high-glucose conditions (20 mM = 360 mg/dL) and cultured in Dulbecco’s Modified Eagle medium supplemented with 10% FBS and antibiotics (penicillin 0.1 μg/μL and streptomycin 0.1 μg/μL). To analyze the efficiency of in vitro efferocytosis, macrophages were primed with 300 ng/mL of Cyp A in high-glucose conditions for 24 h.

### 2.2. In Vitro Efferocytosis Assays

Efferocytosis is a specialized process in which macrophages clear apoptotic cells to maintain homeostasis. In order to determine whether cyclophilin A influences efferocytosis, an in vitro efferocytosis assay was carried out in the presence and absence of Cyp A using labelled macrophages by both flow cytometry and confocal microscopy assay.

### 2.3. Flow Cytometry Assay 

An in vitro efferocytosis assay was performed, as previously described [9]. Primary human coronary aortic SMCs (HCASMCs) were labeled with 20 μM orange CMTMR CellTracker fluorescent probes (C2927; Life Technologies, Waltham, MA, USA) for 1 h, then cultured overnight in serum-free media. Simultaneously, RAW macrophages were labeled with 20 μM green CMFDA CellTracker probe (C7025; Life Technologies, Waltham, MA, USA) for 1 h, then cultured overnight in standard media with serum supplementation. In the morning, apoptotic HCASMCs were induced to undergo apoptosis by treatment with 1 µM Staurosporin for 3 h. Later, they were harvested and manually counted. Apoptotic cells (1 × 10^5^) were then added to the cultured macrophages, and co-culturing was performed for an additional 1.5 h. At that point, all adherent cells were trypsinized and subjected to FACS using a BD FACS caliber (530 nm [FL1] and >575 nm [FL4]), as described in previously published protocols [10]. Cells that were dual-positive for green (phagocyte) and orange (SMC) represented phagocytosed cells. The efferocytosis rate was then defined as the ratio of dual-positive cells (phagocytosed ABs) to orange-positive/green-negative cells (uneaten apoptotic cells).

### 2.4. Confocal Microscopy Assay 

Non-adherent, non-phagocytosed cells, post coculture of macrophages with SMCs, were washed off. The remaining cells were fixed and stained with Hoechst 33258 (Sigma-Aldrich Chemicals Private Limited, Bangalore, India) and analyzed under an inverted fluorescence microscope using NIS-Elements software. The efferocytosis rate was analyzed by counting the number of efferocytosed cells per field.

### 2.5. Cholesterol Efflux Culture Assays Using Cholesterol Efflux Assay Kit (Cell-Based)

Cholesterol efflux (reverse cholesterol transport) is a process whereby intracellular cholesterol is transported from macrophages to extracellular acceptors, such as apoprotein A. Reverse cholesterol transport plays an important role in preventing development of atherosclerosis by reducing accumulation of cholesterol in the wall of arteries. To analyze whether cyclophilin A impairs cholesterol efflux in macrophages, we used a high-throughput cell-based cholesterol efflux assay kit (ab196985) with fluorescently labeled cholesterol, according to the manufacturer’s instructions. Briefly, cultured macrophages were labelled with premixed labeling reagent containing fluorescently labeled cholesterol for 16 h and incubated at 37 °C in a humidified incubator with 5% CO_2_. After overnight incubation, the labeling reagent was removed, and cells were treated with human serum as cholesterol acceptors in DMEM media. Then, the cells were washed and incubated for 5 h in a 37 °C incubator containing 5% CO_2_. After incubation, fluorescence was measured (Ex/Em = 482/515 nm) both calorimetrically and by IVIS spectrum in vivo imaging assay. The cell monolayer was solubilized by adding 100 µL of cell lysis buffer for 30 min at room temperature, and fluorescence was measured again. Cholesterol efflux was calculated by dividing the fluorescence intensity of the media by total fluorescence intensity of the cell lysate after the same treatment and media. The value obtained was multiplied by 100 to obtain % cholesterol efflux. The final % cholesterol efflux was determined by subtracting the % cholesterol efflux obtained for the control. High-density lipoprotein (HDL) was used as cholesterol acceptor for a positive control. For negative control, serum containing DMEM media was used. 

### 2.6. In Vitro Silencing of Cyclophilin A Gene

Macrophage cells were transfected with Cyp A-siRNA using mission siRNA transfection reagent (Sigma-Aldrich Chemicals Private Limited, Bangalore, India) for 48 h at 37 °C. Primers used for Cyp A mRNA target sequence were 5′- TGGTGTTTGGCAAAGTGAAAGAAGGCATGAATATTGTGGAGGCCATGGAGCGCTTTG-3′. The efficiency of silencing was determined by measuring relative mRNA expression in quantitative real-time PCR (ABI Prism 7900HT sequence detection system) [2]. 

### 2.7. Gene-Expression Analysis of ABCA1 mRNA by RT-PCR

ABCA1 can inhibit lipid-laden-foam cell formation by increasing the reverse cholesterol transport of excessive cholesterol from lipid-loaded macrophages and conserve lipid homeostasis in cells. To examine the effects of cyclophilin A on macrophage ABCA1 expression and ABCA1 mediated cholesterol efflux, we next analyzed the gene expression of ABCA1 in cyclophilin A primed macrophages by quantitative real-time PCR using ABI Prism 7900HT sequence detection system. Briefly, RNA isolated from macrophages of different treatments was reverse-transcribed, and real-time PCR was performed using specific primers of ABCA1 and beta 2 microglobulin. The reactions were performed in triplicate in 384-well plates at 50 °C, 2 min; 95 °C, 10 min; 95 °C, 15 s; 58 °C, 1 min; and 72 °C, 0.30 min, for 40 cycles. Ct values calculated from the expression levels of ABCA1 gene was normalized to endogenous cellular beta 2 microglobulin. The primer sequences of ABCA1 and beta 2 microglobulin were:

ABCA1:

Forward primer 5′-TCCACAAGGTATTTTTGCAAGGC-3′

Reverse primer 5′-ACTATGCAGCAATGTTTTTGTGGC-3′

beta-2-microglobulin:

Forward primer 5′-CCAGCGTACTCCAAAGATTCAG-3′

Reverse primer 5′-GTAAGTCAACTTCAATGTCGGATG-3′

### 2.8. Protein Sample Preparation for LC/MS/MS Analysis In Vitro

To investigate the molecular link between Cyp A and known ligand receptors involved in efferocytosis, we sorted dual-positive cells after co culturing using flow cytometry; the dual-positive cells were obtained from the in vitro efferocytosis assay. The phagocytosed dual-positive cells for green (phagocyte) and orange (SMC) were used for LC/MS/MS analysis. Protein lysates were prepared in 0.5% RapiGest TM SF surfactant in 50 mM NH4HCO3 buffer (Waters Corporation, Milford, MA, USA). Total protein content was estimated by Bradford assay. Peptide was produced for each sample (100 µg of protein) using trypsin digestion, followed by centrifugation at 14,000 rpm for 12 min, and the supernatants were collected and stored at −20 °C until LC/MS/MS analysis.

### 2.9. Liquid Chromatography and Mass Spectrometry

Peptide samples were analyzed by using a nano ACQUITY UPLC^®^ system (Waters Corporation, Milford, MA, USA) coupled with a quadrupole-time-of-flight (Q/TOF) mass spectrometer (SYNAPT-G2, Waters Corporation, Milford, MA, USA) controlled by MassLynx4.1 SCN781 software (Waters Corporation, Milford, MA, USA). Peptides eluted from the nano LC were monitored by the SYNAPT^®^ G2 High Definitions MS™ System (HDMSE System Waters Corporation, Milford, MA, USA). Three technical replicate runs were performed for each sample. 

### 2.10. Data Analysis and Bioinformatics

LC/MSE data were analyzed by Protein Lynx Global SERVER™ v2.5.3 (PLGS, Waters Corporation, Milford, MA, USA), which helps both with protein identification and relative quantification. Homo sapiens database from NCBI was used for database search. Protein identification was carried out by setting the parameters as at least one fragment-ion match for each peptide, at least three fragment-ion matches for protein or a minimum of two peptide matches for identification. Precursor and fragment ions were defined by setting the mass tolerance at 10 and 20 ppm, respectively. Oxidation of methionine was chosen as variable modification, and carbamidomethylation of cysteine was chosen as fixed modification. Dataset was normalized by auto-normalization of PLGS. Label-free quantitative analyses were performed. Samples were compared with respect to normalized peak area/intensity of identified peptides. Number of peptides, score, and sequence coverage parameters were identified for each protein. The reference sequence identifications (RefSeq) obtained after PLGS analysis were converted into gene symbols using Biological Database Network (BioDBnet). Database for Annotation, Visualization and Integrated Discovery (DAVID) was used for categorizing gene symbols into different biological functions. Statistical analysis and graphical representations were done using MS-Excel 2013. The molecular link between Cyp A and ligand receptors involved in efferocytosis was explored using protein docking. To understand the topological relationship between Cyp A and ligand receptors, we selected 28 known genes reported in the efferocytosis process. These genes include CALR, MFGE8, CX3CL1, ABCA6, ICAM3, GAS6, APOH, PROS1, C1QB, ANXA1, CD 47, LRP1, MBL2, SIRPA, NR1H3, PPARG, LRPAP1, TGFB1, BAI1, TIMD4, CD14, MERTK, CD36, ELMO1, DOCK1, AKT1, PANX1 and GULP1. The similarity of amino-acid sequences between proteins was analyzed using the Schrodinger platform.

### 2.11. Quantification of CD 47 by Confocal Microscopy

For immunostaining of CD 47, RAW macrophages were incubated with primary antibody of mouse anti-CD 47 antibody (1:100) (Novus Biologicals, CO, USA) at 4 °C overnight, followed by incubation with secondary antibody of Alexa flour 488-conjugated anti-mouse antibody (1:200) (Abcam, Cambridge, UK) for 1 h at room temperature. Cells were counterstained with 10 μg/mL of Hoechst 33258 (Sigma) for 5 min and quantified using NIS-Elements Viewer microscope-imaging software with a 63 × 1.3 numerical aperture oil-immersion lens using dual excitation (488 nm for FITC) and emission (515–540 nm for FITC) filter sets.

### 2.12. Western Blot Analysis

After treatment, cells were lysed in cell lysis buffer containing protease inhibitor cocktail (Sigma-Aldrich). The total cell lysates were loaded on SDS-PAGE and electro-transferred into nitrocellulose membrane, followed by incubation with the appropriate primary antibody at 4 °C overnight. The primary antibodies used were mouse anti-cyclophilin A (ab-58144) (1:1000), mouse anti-β Actin (sc-47778) (1:1000), mouse anti-CD 47 antibody (1:1000) (B6H12.2, Novus Biologicals) and rabbit anti-calreticulin (1:1000) (ab-2907). The membranes were later incubated with specific secondary antibodies: anti-mouse IgG-HRP (Abcam) at a dilution of 1:5000. The proteins were visualized with Clarity Western ECL substrate. The bands were analyzed by Quantity One 1D image-analysis software (Bio-Rad Laboratories, Hercules, CA, USA).

### 2.13. In Vivo Study Model

Further, we used New Zealand white rabbits (NZW) (*n* = 12) to demonstrate the effect of Cyp A on impaired efferocytosis. All animal experiments were performed according to experimental protocols that were approved by the Institute Animal Ethics Committee, RGCB (IAEC/803/SURYA/2018). The animals were categorized into two groups. Group 1 animals (*n* = 6) were fed with a normal diet (ND) containing 16.6% fiber, 14.5% protein and 8.3% mineral ash. Animals of group II (*n* = 6) were fed a high-cholesterol diet (HFD), which consisted of 0.5% (*w*/*w*) cholesterol/kg (5 g cholesterol, 150 g fat/kg rabbit chow), 2.6% sugar and 3% saturated fatty acids. To induce fatty streak formation, the animals were fed with HFD for 12 weeks. All animals had unobstructed admittance to water and were conserved on a 12-hlight–dark cycle in a pathogen-free environment. All animals were observed daily. At the end of the 12 weeks, blood samples were collected, and animals were euthanized by intramuscular injection of a combination of xylazine and thiopental. Aortae were isolated for histopathological analysis. Blood samples were collected from the marginal ear vein, and plasma was prepared and stored at −80 °C. Low-density lipoprotein (LDL), triglycerides and glucose levels in serum were measured using the Biosystems kit (LDL-code no: 11585 and Triglycerides-code no: 11528; Biosystems S.A., Barcelona, Spain), following the manufacturer’s instructions. Plasma Cyp A levels were determined by enzyme-linked immunosorbent assay (ELISA; R&D systems, USCN Life Science Inc., TX, USA), as per the manufacturer’s instructions.

### 2.14. ORO Staining 

Enface staining of rabbit aortae was performed using Oil Red O Staining, as mentioned earlier [4]. Briefly, whole rabbit aortae were collected and fixed on a petri dish with black wax with PBS and cut open vertically to expose the inner area of the aorta, washed and rinsed with 60% isopropanol. Rabbit aortae were stained with ORO stain for 30 min, followed by rinsing with 60% isopropanol for 2 s to remove excess lipids. Lipid-laden plaque area was analyzed by measuring the ratio of ORO-stained plaque areas to plaque area from total aorta within the aortic surface using ImageJ software.

### 2.15. Histological Analysis

Whole aortae were isolated for morphological analysis, fixed for 24 h with 10% phosphate-buffered formalin and embedded in paraffin. Cross-sections of seven microns thickness were prepared, and sections were stained with haematoxylin and eosin, Masson’s trichrome stain or used for immunostaining. Statistical analyses were performed using Image-Pro Plus software. Lesion area distinguished from acellular region on staining was analyzed by microscopy using NIS elements software.

### 2.16. Immunohistochemistry

For histopathological analysis of various proteins, the formaldehyde-fixed paraffin sections of tissue were incubated with primary antibodies, anti α smooth muscle actin (α-SMA) (ab-7817; 1:200; Abcam), cyclophilin A (ab41684; 1:200; Abcam), calreticulin (ab-2907; 1:100; Abcam) and CD 47 (B6H12.2; 1:100; Novus Biologicals) overnight at 4 °C. Species- and isotype-matched IgG was used as negative control. Anti-rabbit IgG-HRP (ab-97051) and anti-mouse IgG HRP (ab-6789) were used as secondary antibodies at a dilution of 1: 400 and 1:200, respectively. Quantitative analysis was performed manually by analyzing diaminobenzidine-tetrahydrochloride- (DAB) positive areas using ImageJ software.

### 2.17. Statistical Analysis

All experiments were performed in triplicate. Variable comparison between two groups was done by Fisher’s exact *t* test. Analysis of variance (ANOVA) using R package, Geisser Greenhouse’s epsilon, was used to analyze the differences among various cell treatments. Continuous variables with normal distribution were expressed as mean ± SD. Data were analyzed using a linear mixed effects model, with the ‘treatment’ (introduction of Cyp A and siRNA) and ‘control’ groups treated as fixed-effect variables. The repetitions were considered as random effects nested within the fixed effect. In vivo data analysis was performed using ImageJ (version 1.45s, Bethesda, MD, USA), and in vitro data were analyzed using Graph Pad Prism (Graph Pad Software, San Diego, CA, USA). *p* < 0.05 was considered statistically significant. 

## 3. Results

### 3.1. In Vitro Assay

#### 3.1.1. Cyclophilin A Impairs Efferocytosis of Apoptotic Macrophages

**Figure 1:**
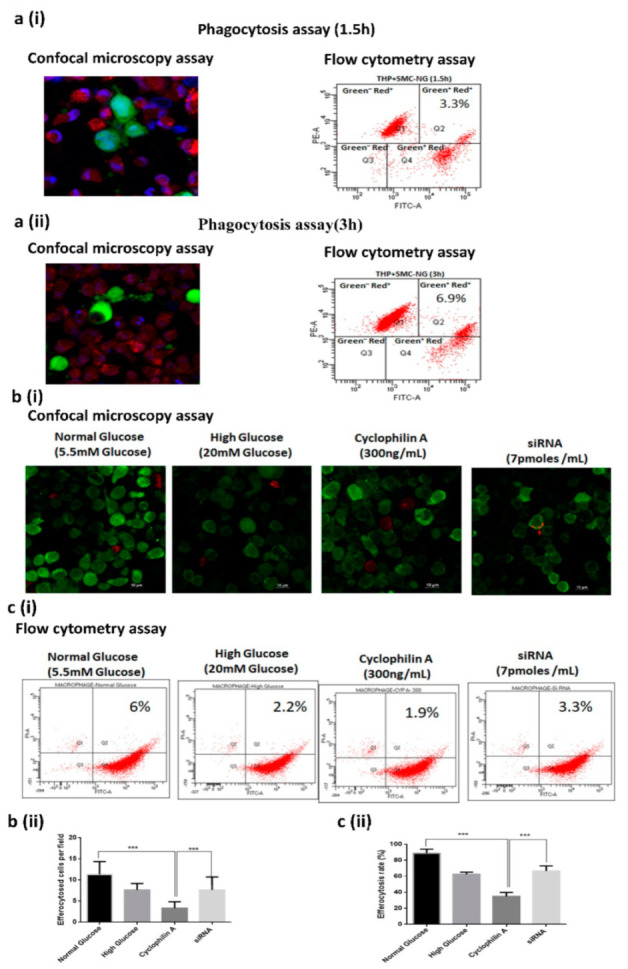
Cyclophilin A impairs efferocytosis of apoptotic macrophages: The effect of Cyp A on the efferocytic capacity of macrophages was determined by in vitro efferocytosis assay. (**a**) (i & ii). In vitro efferocytosis assay using confocal microscopy and flow cytometry assay at 1.5 h and 3 h post-treatment with Cyp A. (**b**) (i & ii). Confocal images, quantitative analysis of confocal images, (**c**) (i & ii). flow cytometry dot plots and efferocytosis rate of phagocytozed cells in the presence of Cyp A. Efferocytosis assay revealed that Cyp A-treated macrophages had a higher percentage of free cell-tracker green-stained apoptotic HCASMCs. Red-colored cells represent healthy phagocytes, and dual-positive cells (both red and green) represent efficient phagocytosis. * *p*< 0.05, *** *p*< 0.001.

The effect of Cyp A on the efferocytic capacity of macrophages was determined by in vitro efferocytosis assay using RAW macrophages and apoptotic human coronary artery smooth muscle cells (HCASMCs). Apoptotic HCASMCs were labeled fluorescent green and cocultured with orange-labeled Cyp A-treated phagocytes. A higher uptake of apoptotic cells was observed in the normal glucose (NG) group within 3 h of coculturing (Figure 1(ai,aii)), whereas reduced engulfment of apoptotic cells was observed in the Cyp A-treated cells (Figure 1b,c). Cyp A-treated macrophages had a higher percentage of green-stained apoptotic HCASMCs when compared to NG controls (31.6% reduction) (Figure 1b,c). Post-silencing of Cyp A by using mission siRNA, the uptake and clearance of apoptotic HCASMCs by macrophages increased to 42.2% (Figure 1b,c) suggesting that Cyp A caused a decrease in efferocytic ability of macrophages.

#### 3.1.2. Cyclophilin A Reduces Cholesterol Efflux of Macrophages

**Figure 2:**
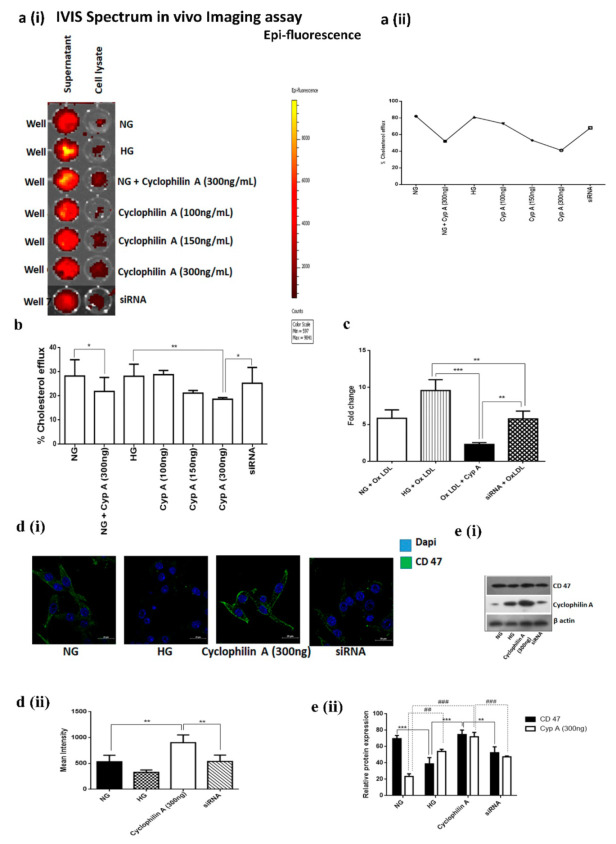
Cyclophilin A reduces cholesterol efflux of macrophages. Cholesterol efflux was measured in an in vitro system using a cell-based cholesterol efflux assay kit after exposing macrophage cells preloaded with labelled cholesterol in the presence of Cyp A to a cholesterol acceptor, such as HDL or human serum. (**a**) (i and ii). Percentage cholesterol efflux was evaluated by in vivo IVIS Spectrum Imaging assay; (**b**) cholesterol efflux in cells was measured using a cell-based cholesterol efflux assay kit. Efflux from Cyp A-treated macrophages was reduced in 5 h (41%), compared to control cells (82%); (**c**) gene-expression analysis of ABCA1 mRNA by RT-PCR. (**d**) (i and ii). Confocal images indicate increased cellular-level expression of CD 47 in Cyp A-treated macrophages, similar to untreated macrophages; * *p*< 0.05 was considered significant. (**e**) (i and ii). The expression of CD 47 and in Cyp A macrophages treated by immunoblotting. * *p*< 0.05, ** *p*< 0.01, *** *p*< 0.001 for CD 47 and **^#^** *p*< 0.05, ^##^ *p*< 0.01 ^###^ *p*< 0.001 for Cyp A was considered significant. The data were compared using a one-way ANOVA, followed by multiple-comparison test using an SNK test.

Macrophages can unload excessive cholesterol and prevent cholesterol accumulation by transferring surplus cholesterol from the plasma membrane of macrophages to adjacent smooth muscle cells. Cholesterol efflux can be measured in an in vitro system by exposing macrophage cells preloaded with labelled cholesterol to a cholesterol acceptor, such as HDL or human serum, in the presence of Cyp A. Efflux was reduced to 41% in 5 h in Cyp A-treated macrophages, whereas in control cells, cholesterol efflux was 82%. Post-siRNA treatment, cholesterol efflux in macrophages was restored to 68% (Figure 2(ai,aii),b).

#### 3.1.3. Cyclophilin A Decreases ABCA1 Expression and ABCA1-mediated Cholesterol Efflux from Macrophage-Derived Foam Cells

Lipid uptake by macrophages and formation of foam cells precede lesion development in the aorta. ABCA1 can inhibit foam-cell formation by transferring excess cholesterol from lipid-loaded macrophages. We studied the effects of Cyp A on ABCA1 expression and ABCA1-mediated cholesterol efflux by treating macrophages with 50 μg/mL ox-LDL for 24 h in the presence of Cyp A. Cyp A is known to induce foam-cell formation. There was a 1.25-fold reduction in ABCA1 mRNA-level expression in macrophages treated with Cyp A when compared to untreated macrophages. Upon silencing of the Cyp A gene, there was a 6-fold increase in expression of ABCA1 mRNA (Figure 2c).

#### 3.1.4. CD 47 Is Overexpressed in Cyclophilin A-Primed Macrophages 

CD 47 is a potent *don’t-eat-me* signal that inhibits macrophage phagocytosis. Cellular-level expression of CD 47 was high in Cyp A-treated macrophages (Figure 2(di,dii)). Concurrently, protein expression of CD 47 was increased in Cyp A-treated macrophages (Figure 2(ei,eii)).

#### 3.1.5. Cyclophilin A Increases Expression of CD 47 and Reduces Expression of Eat-Me Signal, Calreticulin

**Figure 3:**
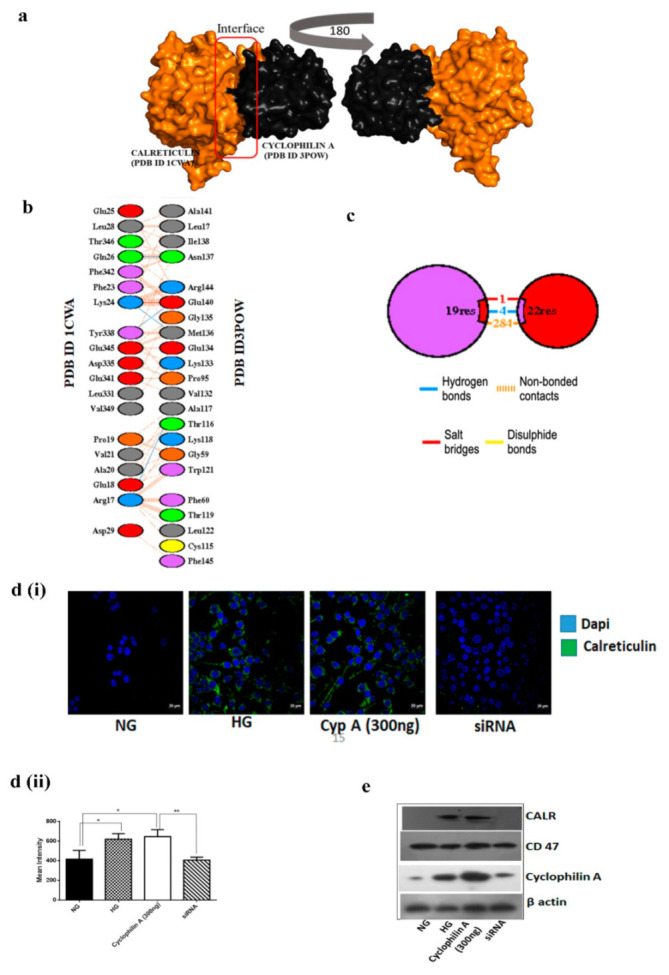
Cyclophilin A is associated with increased expression of CD 47 and is associated with reduced expression of the phagocyte receptor ligand calreticulin. The molecular association between Cyp A and known ligand receptors involved in efferocytosis was analyzed using molecular modeling studies. (**a**). Protein-protein docking analysis. CALR (PDB ID 1CWA) binds with Cyp A (PDB ID 3POW), with a high docking score of −219 and a ligand RMSD value of 51.69 Å. (**b**,**c**). Analysis of amino acids between two candidate proteins, CALR and Cyp A. (**d**) (i and ii). Confocal images indicate an increased cellular-level expression of calreticulin in Cyp A-treated macrophages. (**e**). Western blot images displaying increased CD 47 expression over cell-surface calreticulin in Cyp A-treated macrophages. * *p* < 0.05, ** *p* < 0.01was considered significant. The data were compared using a one-way ANOVA, followed by multiple-comparison test using an SNK test.

We further explored the molecular link between Cyp A and the known ligand receptors involved in efferocytosis. Molecular modeling studies, such as protein-protein interaction, were undertaken. Phagocytosed dual-positive cells were used for LC/MS/MS analysis and around 680 proteins were reported to be upregulated in Cyp A-treated macrophages when compared to untreated macrophages. To understand the topological relationship between Cyp A and ligand receptors for efferocytosis, we selected 28 genes reported in the mechanism of efferocytosis [10]. These genes include CALR, MFGE8, CX3CL1, ABCA6, ICAM3, GAS6, APOH, PROS1, C1QB, ANXA1, CD 47, LRP1, MBL2, SIRPA, NR1H3, PPARG, LRPAP1, TGFB1, BAI1, TIMD4, CD14, MERTK, CD36, ELMO1, DOCK1, AKT1, PANX1 and GULP1. We initially evaluated the average linkage hierarchical clustering by performing protein docking on the reduced topological overlap matrix representing all pairwise links between these 28 genes and Cyp A. Only three genes, namely AnnexinA1 (ANXA1), calreticulin (CALR) and Complement C1q subcomponent subunit B (C1QB), had increased binding affinity with Cyp A. Using protein-protein docking techniques, we observed that CALR (PDB ID: 1CWA), a key phagocyte receptor ligand, binds with Cyp A (PDB ID: 3POW), with a high docking score of −219 and an RMSD (docking root mean square deviation) value of 51.69 Å (Figure 3a). Next, we analyzed the interaction between amino acids of CALR and Cyp A proteins (Figure 3b,c). 

Increased surface expression of CD 47 is considered to protect apoptotic cells from calreticulin-mediated phagocytosis. We hence validated the expression pattern of Cyp A, calreticulin and CD 47 in apoptotic macrophages by immunoblotting and immunofluorescence assays. Cellular-level expression of calreticulin was increased in Cyp A-treated macrophages, whereas it was reduced in silencing of Cyp A (Figure 3(di,dii). There was more CD 47 expression than expression of cell-surface calreticulin in Cyp A-treated macrophages (Figure 3e). Upon silencing of Cyp A, calreticulin expression was dominant over CD 47 expression (*p* < 0.001) (Figure 3e). 

### 3.2. In Vivo Results

CD 47 and Cyclophilin A Levels are increased in Aortic Lesions in High-Fat-Die- (HFD) Fed New Zealand White Rabbits 

We further validated these results in a high-fat-diet-fed (HFD) New Zealand white rabbit model. Aortae of HFD-fed rabbits had extensive areas of intimal lesions comprised of lipid deposits, smooth muscle cells, macrophages and collagen. 

**Figure 4:**
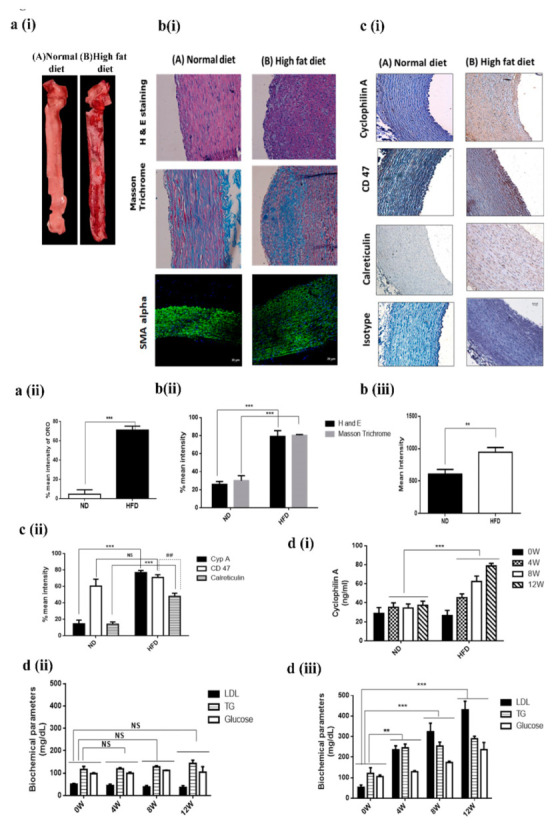
CD 47 and plasma cyclophilin A levels were increased in HFD-fed New Zealand white rabbit. (**a**) (i and ii). ORO staining of rabbit aorta. (**b**) (i and ii). Histological analysis of the cross sections from the aortic arch of HFD- and ND-fed rabbit after staining with H&E staining and Masson Trichrome. (**b**) (i and iii). Confocal images of the expression of α- smooth muscle cell actin (α-SMA) of the cross sections from the aortic arch of HFD- and ND-fed rabbit; (**c**) (i and ii). Immunohistochemical staining of cross sections from the aortic arch of HFD- and ND-fed rabbit after staining with Cyp A, CD 47 and calreticulin. (**d**) Mean values of (i) plasma cyclophilin A level and biochemical parameters in the study groups. (ii) ND group; (iii) HFD group. Levels of plasma Cyp A, along with serum glucose, triglyceride and LDL, were increased in animals fed HFD compared to animals fed ND; ** *p* < 0.01, *** *p* < 0.001 was considered significant (HFD group vs ND group). ## *p* < 0.01was considered significant (CD 47 vs Calreticulin in HFD group). The data were compared using one-way ANOVA, followed by multiple-comparison test using an SNK test.

To evaluate atherosclerotic plaques, we performed an enface staining of rabbit aortae by Oil Red O (ORO) staining. ORO staining demonstrated a significant increase in lesion area (71.35%) in HFD-fed NZW rabbits in relation to ND-fed rabbits (4.76%) (Figure 4(ai,aii)). Aortic lesions in the cross sections of thoracic aorta were quantified using H and E staining. Some of the lesions were acellular, devoid of nuclei and consisting of only a few viable cells (*p* < 0.001) (Figure 4(bi,bii)), suggesting an increased lesion area in the HFD group compared to the ND group. In these lesions, a 54% increase in collagen deposition was observed (Figure 4(bi,bii)), along with increased expression of αSMA in the aortic media of HFD-fed rabbits compared to normal-diet-fed rabbits (ND) (Figure 4(bi,biii)). An increased tissue-level expression of CD 47 was also observed, along with increased expression of Cyp A and decreased calreticulin expression in intimal smooth muscle cells of aortae of HFD-fed rabbits (*p*< 0.001) (Figure 4(ci,cii)). 

Levels of plasma Cyp A (Figure 4(di)), serum glucose, triglyceride and LDL were increased in animals fed a HFD (Figure 4(dii)) when compared with the levels in animals fed an ND (Figure 4(diii)).

## 4. Discussion

Our studies provide a mechanism for impaired efferocytosis in apoptotic macrophages, a known determinant of the severity of atherosclerosis and vulnerability of plaques to rupture. We discovered that cyclophilin A (Cyp A), well-recognized to participate in several mechanisms in the progression of atherosclerosis, can disturb efferocytosis in apoptotic macrophages. Cyp A increases the expression of the *don’t-eat-me* signal, CD 47, and decreases expression of the phagocyte receptor ligand calreticulin in macrophages. Cyp A also reduces ABCA1-mediated cholesterol efflux in foam cells derived from macrophages. Our findings are significant, as Cyp A effects observed in this study could synergize with other mechanisms of Cyp A in acceleration of progression of atherosclerosis.

Efferocytosis involves several sequential events. These include (i) recognition of apoptotic cells by phagocytic receptors, (ii) activation of intracellular signaling cascades and cytoskeletal rearrangements for engulfment of apoptotic cells and (iii) post-engulfment process for phagosome maturation and degradation of apoptotic cells [5]. These mechanisms are regulated by *find-me*, *eat-me* and *don’t-eat-me* signals. Cells that undergo apoptosis express *find-me* signals to attract phagocytes. Dying cells express *eat-me* signals on the cell surface to indicate their readiness to be engulfed by macrophages. The best-characterized *eat-me* signal is phosphatidylserine on the surface of apoptotic cells. Healthy cells display *don’t-eat-me* signals, such as CD 47 and CD31, to avoid efferocytosis. Prompt and efficient clearance of apoptotic cells by phagocytosis is essential to maintenance of tissue homeostasis and prevention of secondary necrosis [6]. 

Apoptotic cells are cleared quickly in almost all tissue beds. However, their removal appears to be significantly impaired in atherosclerotic blood vessels [7]. Macrophages containing abundant apoptotic material are detected in atherosclerotic plaques. Their accumulation is seen in the necrotic core of atherosclerotic lesions, which is a hallmark of atherosclerotic plaques vulnerable to rupture. There is also evidence that efferocytosis occurs in atherosclerosis [8]. In normal tissue, efficient cross talk between apoptotic and phagocytic mechanisms regulates efferocytosis. Cyclophilin A reportedly increases macrophage apoptosis through mitochondria-mediated death-signaling pathways. An 8-fold increase in the number of apoptotic cells in the lesion area of HFD-fed rabbits was observed [4]. Efferocytosis is impaired in atherosclerotic lesions [11].

We observed that CD 47 is overexpressed in apoptotic macrophages [12] in the presence of Cyp A and that this could impede clearance of apoptotic debris by neutralizing the action of calreticulin, a ligand for phagocyte receptors. Clearance of apoptotic cells by macrophages could be increased by mission-siRNA-mediated silencing of the Cyp A gene. 

Cyp A also reduces cholesterol efflux from lesional macrophages, thus promoting foam-cell transformation. Rapid breakdown of accumulated apoptotic cell membranes and release of plaque-destabilizing proteases [11] and pro-inflammatory cytokines [13] can further accelerate atherosclerosis and promote lesion vulnerability [14]. Cyp A levels were found to be high in advanced atherosclerotic lesions of aorta in high-fat diet-fed animals. Thus, our studies indicate that Cyp A, by impairing efferocytosis and increasing macrophage apoptosis, can contribute to the progression of atherosclerotic lesions.

Cyclophilin A is an immunophilin with PPIase enzymatic activity and plays an active role in protein folding [15]. Cyclophilin A is also secreted from several cell types, such as monocyte [2], endothelial cells [16] and vascular smooth muscle cells [17] in both extracellular and intracellular forms in response to stimuli such as high glucose, presence of modified LDL and oxidative stress. Plasma cyclophilin A levels contribute to a pro-inflammatory milieu and trigger vessel-wall inflammation. Overexpression of cyclophilin A in macrophages induces the expression of scavenger receptors, resulting in the formation of lipid-laden foam cells and subsequent lesion formation. Cyclophilin A therefore functions as a cytokine and causes atherosclerotic lesion formation [2]. Plasma Cyp A levels are increased in patients with type 2 diabetes and clinically manifested vascular disease [9]. Recently, we demonstrated that the mechanisms by which Cyp A can contribute to rapid progression of vascular lesions in high glucose conditions involve the induction of foam-cell formation through an increase in oxidative stress [2], increasing the levels of other pro-inflammatory cytokines [2] and increasing macrophage apoptosis [4]. The present finding suggests an additional mechanism.

Macrophage foam cells are abundant in atherosclerotic plaques vulnerable to rupture [18]. Normally, macrophages unload extra cholesterol to apolipoprotein A1 and high-density lipoproteins via an efflux pathway. This is mediated by ATP-binding cassette transporters ABCA1 and ABCG1. Cholesterol efflux modulated by high-density lipoprotein is an atheroprotective mechanism [19,20,21,22,23,24,25,26]. In atherosclerotic lesions, transfer of cholesterol to intimal smooth muscle cells increases cholesterol levels in these cells, converting them into macrophage-like cells [27,28]. They secrete pro-inflammatory cytokines and make atherosclerotic plaques unstable [29]. A series of cell-based cholesterol-efflux experiments in our study indicate that the reverse cholesterol-efflux ability of macrophages is impaired in the presence of Cyp A. 

An important cause of advancement of atherosclerosis is increased apoptosis of cells in lesions. A secondary reason is defective efferocytosis when apoptotic cells are poor substrates for phagocytes [14]. Macrophages are the key effector cells that identify *eat-me, find-me* and *don’t-eat-me* signals. Healthy cells express CD 47, a *don’t-eat-me* signal that protects them from macrophage engulfment [30,31]. CD 47 is consistently upregulated in atherosclerotic plaques in humans, as well as mice [12]. In mice, lesional efferocytosis was improved, and necrotic areas were reduced after administration of CD 47-blocking antibodies [14]. Our results support the view that upregulation of CD 47 in the presence of Cyp A could impair macrophage efferocytosis in atherosclerotic lesions. An increase in apoptotic material in lesions also suggests in adequate efferocytosis [32]. 

Calreticulin, a calcium-binding chaperon, is a well-known *eat-me-signal* or pro-apoptotic molecule expressed on the surface of apoptotic cells. It stimulates phagocytosis and immunogenicity of apoptotic cells, possibly by interfering with the PS–C1q interaction [33]. During efferocytosis, macrophages display several receptors that bind either directly or indirectly, via bridging molecules, to calreticulin. Several studies suggest that apoptotic cells in atherosclerotic lesions express lower amounts of calreticulin [14].

Calreticulin and Cyp A are multifunctional proteins, and their interactions play an important role in several cellular processes [34]. The Ca^2+^ binding site of the P-domain of calreticulin is proline-rich [35]. Cyp A can bind to proline and thus potentially affect Ca^2+^-binding activity of calreticulin by forming a complex [34]. In string analysis of the proteomics data, we observed an association of calreticulin and C1q proteins with Cyp A. We also found that Cyp A can repress the apoptotic effect of calreticulin by upregulating CD 47. We have not confirmed these findings in Cyp A knockout animals. 

## 5. Conclusions

**Figure 5:**
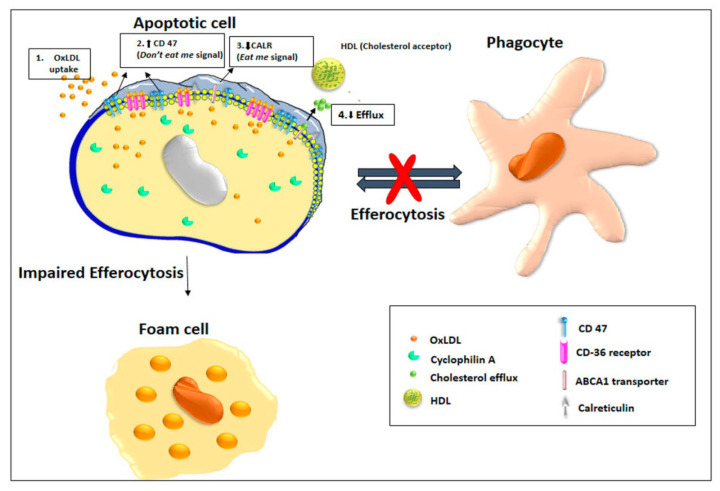
Cyclophilin A impairs efferocytosis by overexpressing *don’t-eat-me* signal, CD 47, and downregulating pro-apoptotic factor calreticulin. Cyclophilin A impairs efferocytosis and promotes atherosclerosis progression by (1) increasing OxLDL uptake through CD36, (2) reducing ABCA1-mediated reverse cholesterol efflux and (3) phagocyte survival by enhancing CD 47 expression (4) over calreticulin-mediated pathways and thereby stimulates fatty streak formation. Inefficient phagocytic clearance of apoptotic cells by lesional macrophages together with increased presence of apoptotic cells in the vascular lumen is the major reason for progression of fatty streaks to advanced atherosclerotic lesions.

In summary, our studies indicate that Cyp A impairs macrophage efferocytosis. A decrease in ABCA1-mediated reverse cholesterol efflux and impaired efferocytosis mediated by enhanced CD 47 expression over calreticulin can lead to progression of atherosclerosis lesions (Figure 5). We have previously reported that Cyp A induces apoptotic cell death through caspase 3-mediated mitochondrial death-signaling pathways [4]. Inefficient phagocytic clearance of apoptotic cells due to impaired efferocytosis is an additional pathway for progression of atherosclerosis caused by Cyp A. Inhibitors of these actions of Cyp A may be of potential use in reducing the vulnerability of atherosclerosis to rupture.

## Data Availability

The data that support the findings of this study are available from the corresponding author upon reasonable request.

## Abbreviations

Cyp A	Cyclophilin A
CD 47	Cluster of Differentiation 47
ABCA1-ATP	ATP-binding cassette transporter A1
NG	Normal Glucose
HFD	High Fat Diet
LDL	Low-density lipoprotein
HG	High Glucose
HCASMC	Human coronary artery smooth muscle cells
FACS	Fluorescence-activated cell sorting
DMEM	Dulbecco’s Modified Eagle Medium
HDL	High-density lipoprotein
NCBI	National Center for Biotechnology Information
NZW	New Zealand White Rabbit
CALR	Calreticulin
C1QB	Complement C1q B Chain
ANXA1	Annexin-A1
siRNA	Small interfering RNA
